# A High-Precision Method for Extracting Lateral Deformation in Operational Shield Tunnels Based on LiDAR Point Cloud Analysis

**DOI:** 10.3390/s26103111

**Published:** 2026-05-14

**Authors:** Sijia Tang, Xiangyang Xu

**Affiliations:** School of Rail Transportation, Soochow University, Suzhou 215006, China; 20224246030@stu.suda.edu.cn

**Keywords:** LiDAR point clouds, semantic segmentation, elliptical fitting, shield-driven tunnels, lateral deformation monitoring

## Abstract

Deformation monitoring is critical for structural health assessment of operational shield tunnels in urban rail transit. LiDAR point clouds in operating tunnels usually contain auxiliary facilities, occlusions, noise, and uneven point density. Conventional section-by-section ellipse fitting often leads to unstable parameter jumps between adjacent sections. This paper presents a high-precision method to extract lateral deformation from tunnel LiDAR point clouds. First, a point-wise attention Transformer network (PWAT) is proposed based on PointNet++ for lining segmentation, using k-NN adaptive sampling, geometric position encoding, and geometry-constrained multi-head self-attention. Second, a continuity-constrained RANSAC (CC-RANSAC) algorithm is developed to improve ellipse parameter stability by adding continuity penalties between neighboring sections. Experiments were carried out on a Shanghai metro shield tunnel. Results show that PWAT achieves 99.53% overall accuracy and 99.06% mIoU in six-class segmentation. CC-RANSAC reduces the mean residual to 2.0 mm and the center jump rate to 4.2%. Compared with total station data, the mean absolute error and root mean square error are 1.35 mm and 1.68 mm. The proposed method can automatically and accurately extract lateral deformation for operational shield tunnels.

## 1. Introduction

With the rapid development of urban rail transit, shield tunnels are widely used in urban underground transportation [1,2]. During long-term operation, tunnels are affected by ground load, groundwater, nearby construction, and material aging. These factors cause lateral convergence, section elliptical deformation, stress concentration, and uneven deformation. Lateral deformation directly reflects the stress state and geometric condition of the tunnel cross-section, and is a key index for structural safety evaluation [3]. Therefore, achieving high-precision, automatic, and continuous lateral deformation detection has become an important task in tunnel health monitoring and intelligent maintenance.

Traditional methods for detecting lateral deformation in tunnels primarily rely on discrete measurement techniques such as convergence gauges and total stations [4,5]. While these methods offer high single-point measurement accuracy, they suffer from limitations such as a limited number of measurement points, high reliance on manual labor, low efficiency, and an inability to capture continuous three-dimensional deformation characteristics across the entire cross-section. Nevertheless, owing to their sub-millimeter single-point accuracy and their well-established role in tunnel deformation monitoring practice, total station measurements are widely adopted as the reference benchmark for validating the absolute accuracy of emerging non-contact monitoring techniques. In contrast, LiDAR can rapidly acquire high-density 3D point clouds of the entire tunnel cross-section in a non-contact manner, providing a rich data foundation for structural contour extraction, cross-section reconstruction, and continuous deformation analysis [6,7].

However, point clouds from operational shield-driven tunnels typically contain complex objects such as lining structures, tracks, power grids, pipelines, wires, and bolt holes, and are accompanied by issues such as occlusion, local data gaps, noise interference, and uneven point density, making them difficult to use directly for high-precision deformation analysis [8]. Therefore, it is essential to first accurately segment the lining point cloud from these complex scenes.

In recent years, deep learning models such as PointNet [9], PointNet++ [10], DGCNN [11], RandLA-Net [12], and Point Transformer have made significant progress in point cloud semantic segmentation tasks [13]. PointNet achieves end-to-end modeling of point cloud processing by directly learning features from unordered point sets [14]; PointNet++ further introduces hierarchical sampling and local feature extraction mechanisms to address PointNet’s insufficient modeling of local context [15]; DGCNN enhances the modeling of local neighborhood relationships through dynamic graph edge construction [16]; RandLA-Net improves the efficiency of large-scale point cloud processing via random sampling and a lightweight architecture [17]; Point Transformer utilizes self-attention mechanisms to enhance the modeling of spatial relationships in point clouds [18]. More recently, several advanced architectures have further pushed the state of the art in point cloud segmentation. PointNeXt revisits the PointNet++ framework with improved training strategies and inverted residual connections, achieving competitive performance with significantly reduced complexity [19]. PTv2 (Point Transformer V2) introduces grouped vector attention and partition-based pooling to improve both accuracy and scalability [20]. OctFormer adopts octree-based window attention to enable efficient processing of large-scale point clouds [21]. GEPT-Net specifically targets tunnel lining segmentation by integrating geometry-enhanced features into a Point Transformer backbone [22]. However, most of the aforementioned methods are designed for indoor or natural scenes and still face certain limitations in structured industrial underground environments. First, point clouds of shield-driven tunnels exhibit significant local density variations [23,24,25]; fixed-radius spherical queries tend to introduce redundant background points in dense areas, while in sparse areas they may fail to adequately capture the complete local structure. Second, features such as bolt holes, electrical wires, and small pipes account for a small proportion of the point cloud and have weak boundaries, requiring higher-resolution geometric perception capabilities [26,27,28,29]; Furthermore, existing models still lack sufficient explicit modeling of regular geometric priors, making it difficult to reliably distinguish between the lining and ancillary facilities at complex boundary locations [30].

In addition to semantic segmentation, the recovery of lateral deformation parameters also poses challenges. A common method involves sequentially extracting cross-sections perpendicular to the tunnel axis, projecting them onto a two-dimensional plane, and performing elliptical fitting [31,32]. Least Squares (LS) method are sensitive to outliers, and while RANSAC offers good robustness [33,34,35], it typically processes each cross-section independently, ignoring the continuous structural features distributed along the tunnel axis. This approach is prone to parameter jumps under conditions of local occlusion and sparse point clouds, affecting the stability of continuous deformation analysis [36,37].

Based on this, this paper proposes a high-precision method for extracting lateral deformation from LiDAR point clouds in complex operational shield tunnel scenarios. This method focuses on the extraction of lining structures and the recovery of continuous cross-sectional parameters from complex tunnel point clouds. First, an improved semantic segmentation network is used to achieve precise extraction of the lining point cloud, then combines elliptical fitting with continuity constraints to recover cross-sectional geometric parameters, and further calculates lateral deformation indicators such as major and minor axes and ellipticity, thereby establishing a complete technical workflow that spans from raw point cloud acquisition to the quantification of structural status.

The main contributions of this paper are as follows:

1. A point-wise attention Transformer network (PWAT) semantic segmentation network is proposed for tunnel point clouds. To handle auxiliary facilities, small objects, and uneven density, the network improves PointNet++ using k-NN adaptive sampling, geometric position encoding, and geometrically constrained multi-head self-attention, in which local spatial offset vectors (point-to-centroid displacements) are encoded as geometric priors and injected into the attention scoring function. It can accurately distinguish tunnel linings from other structures in complex environments.
2. A continuity-constrained RANSAC (CC-RANSAC) algorithm is presented for continuous section ellipse fitting. The method keeps RANSAC’s robustness to outliers and adds geometric continuity constraints between adjacent sections into the scoring function. It reduces parameter jumps and improves the stability of ellipse center and axis recovery.
3. An integrated framework of “semantic segmentation—continuous fitting—deformation analysis” is built. By combining PWAT and CC-RANSAC, the framework realizes automatic processing from raw LiDAR point clouds to lateral deformation indicators. The performance is verified using real metro tunnel data.

## 2. Materials and Methods

### 2.1. Overall Framework

**Figure 1:**
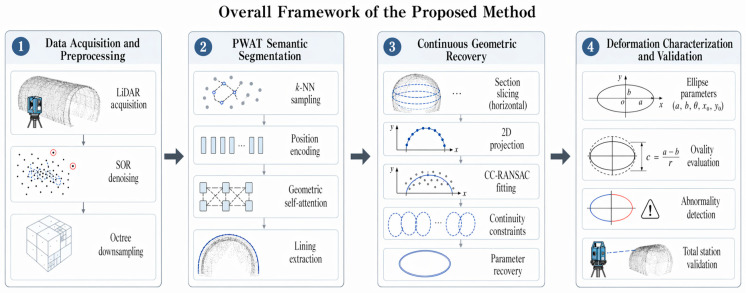
Overall framework of the proposed method.

This paper presents an integrated framework for high-precision lateral deformation extraction from shield tunnel LiDAR point clouds (Figure 1). The framework includes four steps: data acquisition and preprocessing, PWAT semantic segmentation, continuous geometric recovery, and deformation characterization and validation. First, raw LiDAR data is collected and preprocessed using statistical outlier removal (SOR) and octree downsampling to improve data quality and efficiency. Then, the PWAT network is used to segment the lining point cloud from complex background structures using k-NN adaptive sampling, position encoding, and geometric self-attention. Once the lining is extracted, the continuous geometric recovery stage is initiated. The lining point cloud undergoes horizontal section slicing and 2D projection, after which a Constrained and Continuous RANSAC (CC-RANSAC) algorithm is applied to fit the cross-sectional profiles. The continuity constraints imposed in CC-RANSAC consist of two components: a spatial distance constraint $P_{c}$ that limits the offset of ellipse centers between adjacent sections, and a parametric constraint $P_{a}$ that restricts variations in the semi-major axis. Together, they form the penalty term in the composite scoring function to suppress both center jumps and abrupt changes in axis length, thereby ensuring stable and consistent parameter estimation across consecutive sections. Finally, in the deformation characterization and validation stage, the recovered ellipse parameters—including the major and minor axes (a,b), rotation angle (θ), and center coordinates $(x_{0},y_{0})\;$ are utilized to evaluate the ovality ($c=\frac{a-b}{r}$) and detect structural abnormalities. The reliability of the entire proposed methodology is ultimately validated against conventional total station measurements, forming a complete, automated, and highly robust pipeline for tunnel structural health monitoring.

### 2.2. LiDAR Data Acquisition and Preprocessing

#### 2.2.1. LiDAR Data Acquisition in Operational Shield Tunnels

**Figure 2:**
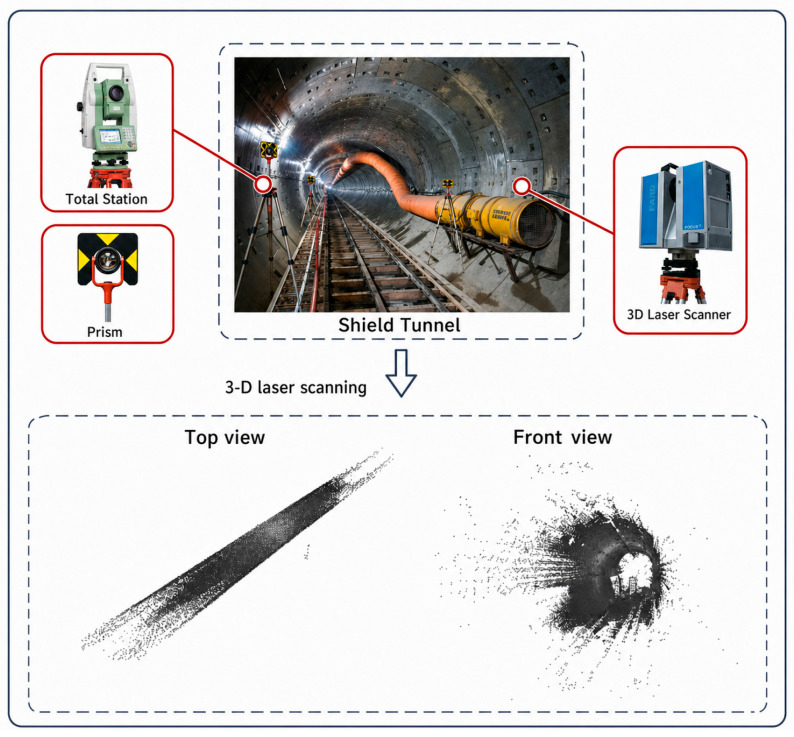
Schematic Diagram of LiDAR Data Collection for Operating a Shield Tunnel.

This study utilized a Leica ScanStation P40 3D laser scanner (Leica Geosystems AG, Heerbrugg, Switzerland) to acquire high-density point cloud data of an operational metro shield tunnel. The measured data was collected from a typical section of an operational metro shield tunnel in Shanghai, covering both straight and curved sections, with a total of approximately 40.44 million points in the raw point cloud. In this study, a 360° panoramic scanning mode was employed for data acquisition. The horizontal and vertical angular resolutions were both set to 0.036°, producing a nominal point spacing of approximately 6.3 mm at a typical scanning distance of 10 m. The scanner operated at a point rate of 1,000,000 points per second, with a single-station scanning time of approximately 3 min. As shown in Figure 2, 3D laser scanning equipment was used to collect full-cross-section point cloud data of the operational shield tunnel, capturing raw point cloud data that includes structures such as the lining, tracks, power supply network, pipelines, and bolt holes. Compared to traditional discrete point measurement methods, LiDAR scanning provides higher-density, more continuous spatial geometric information, offering a reliable data foundation for subsequent semantic segmentation and lateral deformation analysis. Since the raw data contains multiple object types—including lining, tracks, power supply facilities, pipelines, wires, and bolt holes—and is accompanied by some noise and local data gaps, preprocessing is required before it can be used for further analysis.

#### 2.2.2. Statistical Outlier Removal (SOR) Denoising

Due to the complex tunnel environment, outliers and local noise points are commonly found in raw LiDAR point clouds. On the one hand, these points interfere with the deep learning network’s extraction of local structural features; on the other hand, they introduce geometric deviations during the cross-section fitting stage, thereby reducing the accuracy of elliptical parameter recovery. To address this, this paper employs the Statistical Outlier Removal (SOR) algorithm to perform denoising on the point cloud. Let the point cloud dataset be $P=\left\{p_{1},p_{2},\ldots ,p_{n}\right\}$. For any point $p_{i}$, we first calculate the average distance $\mu_{i}$ between $p_{i}$ and the k nearest points in its neighborhood:

(1) $$\mu_{i}=\frac{1}{k}\overset{k}{\underset{j=1\;}{\sum }}\|p_{i}-p_{ij}\|$$

where $\mu_{i}$ is the average distance from point $p_{i}$ to its neighbors, k is the number of nearest neighbors, $p_{ij}$ represents the j-th nearest neighbor of $p_{i}$, and ∥⋅∥ denotes the Euclidean distance.

Next, calculate the global mean μ and standard deviation σ of the average neighborhood distances for all points. If the average neighborhood distance $\;\mu_{i}$ of a point exceeds the threshold μ + αs, that point is identified as an outlier and removed. In this study, statistical filtering is employed to enhance point cloud quality. The number of nearest neighbors k is set to 20 and the standard deviation multiplier threshold α to 2.0, thereby removing noise while retaining the original structural details to the maximum extent.

#### 2.2.3. Octree-Based Voxel Downsampling

**Figure 3:**
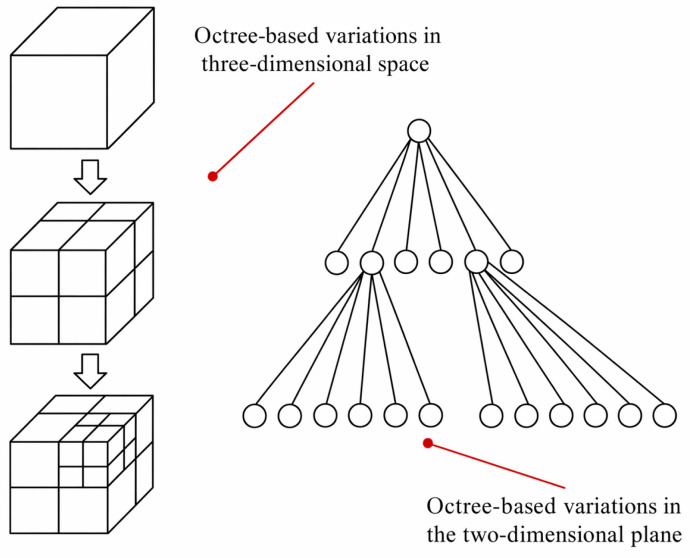
Schematic Diagram of an Octree Structure.

Considering that, for deep learning-based point cloud semantic segmentation tasks, excessively high point cloud density not only significantly increases the burden on video memory and computational resources but may also introduce a large number of redundant background points, thereby affecting the model’s efficiency in learning key local structures. To balance the preservation of geometric details with processing efficiency, this paper employs an octree-based voxel downsampling method to compress point clouds. An octree is a typical hierarchical partitioning structure in three-dimensional space that recursively divides the point cloud space into eight subregions and selects a centroid or representative point within each voxel unit to replace the original point set. Voxel-based downsampling based on this structure can significantly reduce the number of points while effectively preserving the overall geometric contour of the point cloud. Figure 3 shows a schematic diagram of the octree structure.

In the experiment, this paper conducted a comparative analysis of different voxel resolutions and determined the parameters based on a comprehensive evaluation of segmentation accuracy and processing efficiency. When the voxel size is too small (e.g., 0.01 m or 0.02 m), although geometric details are preserved relatively well, the point cloud remains large, resulting in a significant increase in training time and computational resource consumption; when the voxel size is too large (e.g., 0.50 m or larger), fine structures such as bolt holes and joints become noticeably blurred or even disappear, thereby affecting the accuracy of subsequent semantic segmentation and cross-section fitting.

**Figure 4:**
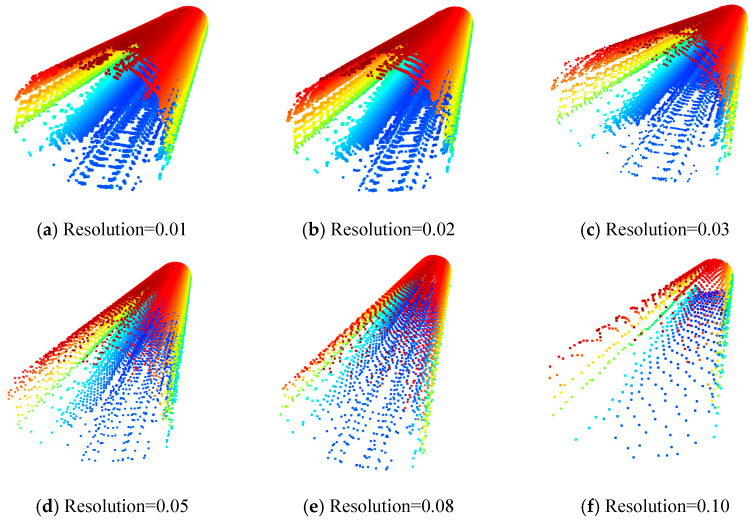
Point cloud compression results for the tunnel section.

As shown in Figure 4, due to the large volume of the initial point cloud data, the transition in compression ratio from 0% to approximately 90% is relatively smooth, and the overall point cloud texture remains clearly visible even as the data volume gradually decreases. When OctreeResolution = 0.08 and the compression ratio is around 99.99%, the point cloud begins to exhibit voids and local errors, resulting in a significant drop in compression quality; whereas when OctreeResolution = 0.10, the point cloud becomes noticeably sparse, and the compression quality is very poor. Overall, while effectively meeting the quality requirements for subsequent data processing, the point cloud compression ratio can be controlled between 99.70% and 99.98%, significantly reducing storage space and achieving a balance between data processing efficiency and quality. For specific compression results, please refer to Table 1.

Comprehensive analysis shows that 0.03 m achieves the best balance between accuracy and efficiency, and is thus adopted as the final resolution in this study. This setting significantly reduces the data volume while preserving key geometric features such as pipe-to-slab joints and bolt holes, thereby providing a data foundation that balances accuracy and efficiency for subsequent PWAT training and CC-RANSAC fitting. The diameter of the bolt holes in the shield tunnel segments of the Shanghai Metro is approximately 60–70 mm. After voxel sampling at a resolution of 0.03 m, 2–3 concentric rings of point clouds can usually still be retained in each bolt hole area, and its circular contour features can be recognized by the network. For different tunnel types with significantly smaller bolt holes, it is recommended to appropriately reduce the voxel resolution (e.g., to 0.01–0.02 m).

### 2.3. PWAT Network for Tunnel Point Cloud Semantic Segmentation

#### 2.3.1. Motivation for PWAT

**Figure 5:**
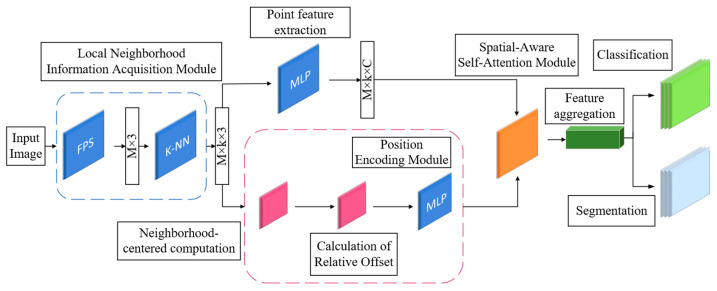
Schematic Diagram of the PWAT Network Architecture.

Point cloud semantic segmentation is a critical step in the LiDAR data processing pipeline for operational shield-driven tunnels, with the goal of accurately extracting lining structures from complex mixed scenes. While PointNet++ performs well in general point cloud segmentation tasks, it still has limitations when directly applied to shield tunnel scenarios: fixed-radius spherical queries struggle to adapt to uneven point cloud density, leading to the loss of features from small targets; max-pooling fails to highlight key boundary points; and there is insufficient utilization of regular geometric priors. To address these issues, this paper proposes the PWAT network to enhance the modeling capabilities of local geometric structures and spatial relationships in complex tunnel point clouds. By introducing a k-nearest-neighbor adaptive sampling strategy, a geometric position encoding module, and a multi-head self-attention mechanism that integrates geometric constraints, PWAT enhances the modeling of local geometric structures and spatial relationships in complex tunnel LiDAR point clouds, thereby improving the classification accuracy of the lining and ancillary facilities. While each of these components—k-NN adaptive sampling, geometric position encoding, and geometry-constrained multi-head self-attention—has been explored individually in prior work, their specific integration within PWAT is designed to address a unique combination of challenges inherent to operational shield tunnel point clouds: simultaneous density non-uniformity, small-object scarcity, and the presence of regular geometric priors from tunnel structures. Unlike general-purpose transformer-based methods such as Point Transformer, which apply self-attention globally without domain-specific geometric constraints, PWAT explicitly encodes local spatial offsets into the attention weight computation (Equation (6)), enabling the model to exploit the cylindrical geometric regularity of tunnel linings as a prior. This targeted co-design of the three modules, rather than any single component in isolation, constitutes the primary methodological novelty of PWAT. The overall architecture of the PWAT network is shown in Figure 5.

#### 2.3.2. K-Nearest-Neighbor Adaptive Sampling

In complex shield tunneling scenarios, there are significant variations in local point cloud density. The traditional PointNet++ uses a fixed-radius ball query to construct local neighborhoods. However, tunnel point clouds exhibit significant density non-uniformity, with dense clusters in areas with smooth tunnel segments and sparse clusters at edges and in occluded regions. A fixed radius is prone to causing feature loss in sparse areas. PWAT introduces an adaptive sampling strategy based on Euclidean distance k-nearest neighbors (k-NN). For a central point $x_{i}$, the neighborhood is constructed by selecting the k nearest points in ascending order of distance:

(2) $$d_{ij}={\left\|x_{i}-x_{j}\right\|}^{2}$$

where $d_{ij}$ is the squared Euclidean distance, $x_{i}$ represents the spatial coordinates of the central point, and $x_{j}$ represents the coordinates of the j-th neighboring point.

The local neighborhood is formed by selecting the k nearest points in ascending order of distance. This strategy adapts the receptive field based on the local point cloud density, suppressing redundant background in high-density regions while preserving more useful structural information in sparse regions.

#### 2.3.3. Geometric Position Encoding

Although the types of ancillary facilities inside tunnels are diverse, they generally follow clear local geometric patterns. If we rely solely on traditional point feature encoding, it is difficult for the model to fully leverage these geometric priorities. To enhance the network’s ability to perceive spatial structures, this paper designs a geometric position encoding module that explicitly models the offset of neighborhood points relative to local centers. By calculating the spatial offset of neighborhood point $x_{i}$ relative to the local neighborhood center point $x_{\mathrm{centroid}}$, and mapping it to high-dimensional position features via a multi-layer perceptron (MLP),

(3) $$P_{i}^{encoded}=\mathrm{MLP}\left(x_{i}-x_{centroid}\right)$$

where $P_{i}^{encoded}$ is the output high-dimensional geometric position feature, MLP denotes the Multi-Layer Perceptron operation, $x_{i}$ is the coordinate of the neighborhood point, and $x_{centroid}$ is the geometric center coordinate of the k-nearest-neighbor local neighborhood centered at $x_{i}$. This module maps coordinates relative to high-dimensional embedding positions, which helps enhance the network’s ability to capture local spatial distribution patterns.

#### 2.3.4. Multi-Head Self-Attention with Geometric Constraints

Traditional PointNet++ uses max-pooling to aggregate local features, and the importance of all neighboring points is not explicitly distinguished during the feature fusion stage. This approach has significant limitations when applied to tunnel point clouds characterized by blurred boundaries, sparse small objects, and complex structures. To address this, this paper introduces a multi-head self-attention mechanism to achieve adaptive weighted aggregation by learning the feature correlations among neighboring points. For a local feature matrix F, a linear mapping is used to generate a query vector Q, a key vector K, and a value vector V:

(4) $$Q=FW_{Q},K=FW_{K},V=FW_{V}$$

where Q, K, and V represent the Query, Key, and Value matrices, respectively; F is the input local feature matrix; and $W_{Q}$, $W_{K}$, and $W_{V}$ are the corresponding learnable linear transformation weight matrices.

The standard self-attention mechanism calculates weights by computing the dot product between the query and the key:

(5) $$A_{ij}=Softmax\left(\frac{q_{i}^{T}k_{j}}{\sqrt{d}}\right)$$

where $A_{ij}$ represents the attention weight between the i-th query and the j-th key, $q_{i}$ and $k_{j}$ are the query and key vectors for the respective points, d is the feature dimension, and Softmax is the normalized exponential function.

To further incorporate geometric priors into the calculation of attention weights, this paper integrates the geometric position encoding $P_{i}^{encoded}$ obtained in the previous section into the attention representation, thereby constructing a geometric constraint-based self-attention mechanism:

(6) $$A_{ij}=Softmax\left(\frac{q_{i}^{T}k_{j}-P_{i}^{encoded}}{\sqrt{d}}\right)$$

The geometric constraint here takes the form of local spatial offset vectors—the displacement between each neighboring point and the k-NN neighborhood centroid—encoded via MLP into high-dimensional position features (Equation (3)) and subtracted from the dot-product attention score. This implicitly encodes local surface geometry such as inter-point distance distribution and neighborhood shape, without requiring explicit surface normals or curvature computation, and biases the attention toward geometrically consistent neighbors within the local tunnel structure. It helps the model better identify complex boundaries, local structures, and small targets. Furthermore, this paper employs a multi-head attention mechanism, using multiple independent attention heads to model local relationships in different subspaces in parallel. Different attention heads can focus on information such as local geometric shapes, spatial topological relationships, and boundary differences, respectively. Finally, the results from each head are concatenated and linearly fused to obtain a richer and more stable representation of local features.

### 2.4. Continuous Geometric Recovery for Deformation Extraction

#### 2.4.1. Slice-Wise Cross-Section Extraction and 2D Projection

**Figure 6:**
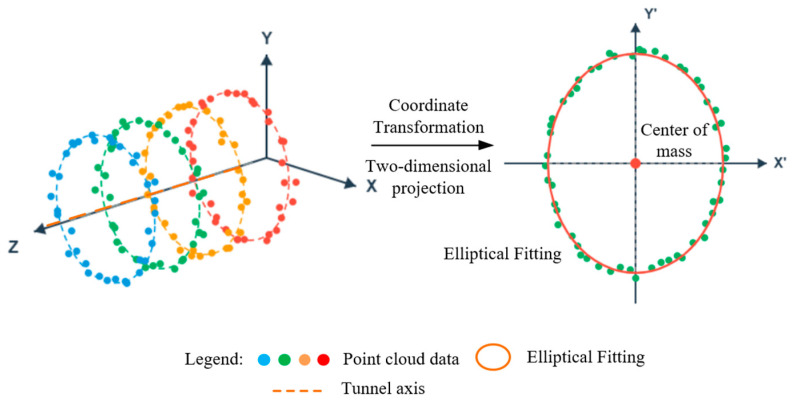
Schematic Diagram Illustrating the Principle of Point Cloud Slicing for Shield Tunnels.

After obtaining the lining point cloud, it is necessary to further extract the parameters of the tunnel’s lateral deformation. Given that shield-driven tunnels extend continuously along the axial direction, this paper first sequentially extracts perpendicular cross-sections along the tunnel’s centerline at equal intervals, and projects each section onto a local two-dimensional plane to construct a set of cross-sectional points for elliptical fitting. The principle of point cloud slicing is illustrated in Figure 6.

**Figure 7:**
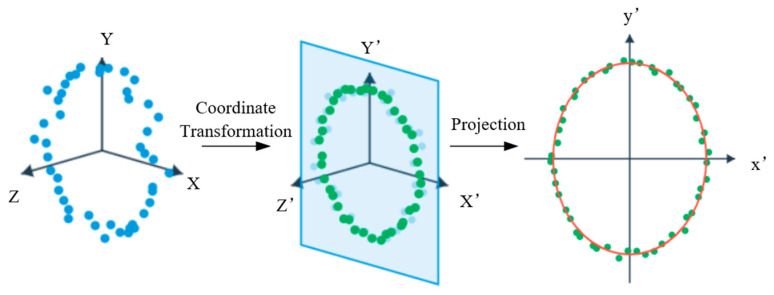
Schematic diagram of the cross-section point cloud projection process.

Let the 3D lining point cloud be denoted as $P\;=\;\left\{p_{i}=\;\left(x_{i},\;y_{i},\;z_{i}\right)\right\}.$ Slices are taken along the axial direction at intervals of 0.1 m, with a slice thickness of Δ = 0.02 m. For the kth slice, its point cloud subset can be represented as all points located near the center of the slice. Considering that the tunnel axis is not strictly parallel to the global coordinate axes under actual conditions, this paper employs principal component analysis (PCA) of the local point cloud to estimate the local axial direction of the slice and construct a local coordinate system based on this. The 3D slice point cloud is then projected onto a local 2D plane orthogonal to the axial direction, yielding a cross-sectional point set for subsequent elliptical fitting. The process of constructing the local coordinates for the cross-section point cloud and the two-dimensional projection is shown in Figure 7.

#### 2.4.2. Traditional RANSAC and Its Limitation for Continuous Sections

**Figure 8:**
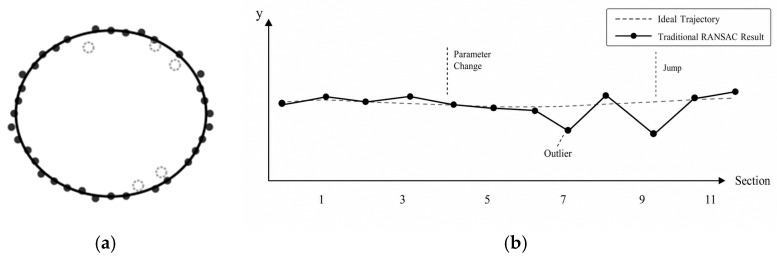
Schematic illustration of the limitations of traditional RANSAC for continuous cross-section fitting: (**a**) independent per-section fitting principle of traditional RANSAC; (**b**) non-physical parameter jumps (center/axis discontinuities) between adjacent sections caused by occlusion and sparse points.

Elliptical fitting is a critical step in lateral deformation analysis. Traditional RANSAC (Random Sampling Consensus) algorithms typically treat each cross-section as an independent sample, without accounting for the structural characteristics of shield tunnels that are distributed continuously along the axial direction. In actual tunnel LiDAR point clouds, local cross-sections may be affected by residual points from ancillary facilities, sparse point clouds, and occlusion-induced data gaps, causing the optimal ellipse solutions for certain cross-sections to deviate from the true geometric state. When these cross-sections are processed independently, parameters such as the ellipse center, major and minor axes, and rotation angle are prone to non-physical jumps, thereby disrupting the continuous deformation pattern along the axial direction. As illustrated in Figure 8a depicts the principle of traditional RANSAC, which performs independent fitting on each cross-section without inter-section coupling, while (b) further demonstrates the resulting parameter discontinuities between adjacent cross-sections under conditions of local occlusion and sparse points. Therefore, it is necessary to introduce geometric continuity constraints between adjacent cross-sections while retaining RANSAC’s outlier rejection capability, thereby enhancing the stability and physical consistency of geometric reconstruction for continuous cross-sections.

#### 2.4.3. Continuity-Constrained RANSAC (CC-RANSAC)

To address the issue of parameter discontinuities in traditional RANSAC when processing consecutive sections, this paper proposes the Continuity-Constrained Random Sampling Consensus Algorithm (CC-RANSAC). Let the elliptical parameters of the kth section be denoted as:

(7) $$\theta_{k}=\left(x_{c,k},y_{c,k},a_{k},b_{k},\phi_{k}\right)$$

Here, $x_{c,k}$ and $y_{c,k}$ are the coordinates of the ellipse center, $a_{k}$ and $b_{k}$ are the major and minor semi-axes, respectively, and $\phi_{k}$ is the rotation angle of the ellipse’s major axis.

For subsequent sections, a continuity penalty term relative to the previous section is introduced on top of the traditional RANSAC in-point rate. For example, the center offset penalty and the major axis variation penalty can be written as:

(8) $$P_{c}\left(\theta^{*}\right)=\max\left(0,\frac{∥c^{*}-c_{k-1}{∥}_{2}-\delta_{c}}{\delta_{c}}\right)$$

(9) $$P_{a}\left(\theta^{*}\right)=\max\left(0,\frac{|a^{*}-a_{k-1}|-\delta_{a}}{\delta_{a}}\right)$$

where $P_{c}\left(\theta^{*}\right)$ and $P_{a}\left(\theta^{*}\right)$ denote the penalty terms for the center offset and major axis variation, respectively; $\theta^{*}$ represents the parameters of the current candidate elliptical model; $c^{*}$ and $a^{*}$ are the center coordinates and major semi-axis of the candidate model; $c_{k-1}$ and $a_{k-1}$ are the fitted center coordinates and major semi-axis of the previous (k−1)-th cross-section; $\delta_{c}$ and $\delta_{a}$ are the predefined allowable tolerance thresholds for center shift and major axis variation, respectively; and $∥\cdot {∥}_{2}$ denotes the Euclidean distance (L2 norm).

The composite score function is:

(10) $$J_{k}\left(\theta^{*}\right)=\eta_{k}\left(\theta^{*}\right)-\alpha P\left(\theta^{*}|\theta_{k-1}\right)$$

Here, $\eta_{k}\left(\theta^{*}\right)$ represents the internal point rate of the candidate model at the current cross-section, $P\left(\theta^{*}\theta_{k-1}\right)$ is the continuity penalty term, and α is the penalty coefficient. The key parameters of CC-RANSAC were determined as follows. The continuity tolerance thresholds $\delta_{c}$ and $\delta_{a}$ are set to 15 mm and 10 mm, respectively, consistent with the maximum physically plausible inter-ring center displacement and semi-major axis variation reported for normally operating Shanghai metro shield tunnels [32,36], and complying with the convergence deformation limits specified in the Chinese metro design standard. Setting these thresholds below these bounds would over-constrain the fitting in sections with genuine structural deformation, while values significantly above them would fail to suppress non-physical parameter jumps between adjacent sections. The penalty coefficient α is set to 0.5 based on empirical tuning on the validation dataset. This value provides a balanced regularization strength: a smaller α would insufficiently suppress inter-section parameter jumps, while a larger α risks over-constraining the fitting and masking genuine structural deformation signals. The inlier ratio threshold $\eta_{min}$ for the reset mechanism is set to 0.3, following the convention established in the original RANSAC framework and consistent with reliability criteria adopted in prior tunnel cross-section fitting studies, below which a fitted model is considered statistically unreliable due to severe occlusion or point sparsity.

To prevent error propagation in the sequential Markovian structure of CC-RANSAC, a reset mechanism is introduced into the algorithm. After each section is fitted, the inlier ratio $\eta_{k}$ of the current best-fit model is evaluated against a predefined reliability threshold $\eta_{min\;}$ (e.g., 0.3). If $\eta_{k}<\eta_{min}$ current section is considered unreliable due to severe occlusion or fitting failure. In this case, the algorithm breaks the Markovian chain: the parameters of the previous section are discarded as the prior reference, and the fitting of the current section is re-initialized using standard independent RANSAC without any continuity constraint. This mechanism effectively interrupts the propagation of accumulated errors along the tunnel axis, ensuring that a poorly fitted section does not bias the recovery of subsequent cross-sections.

**Figure 9:**
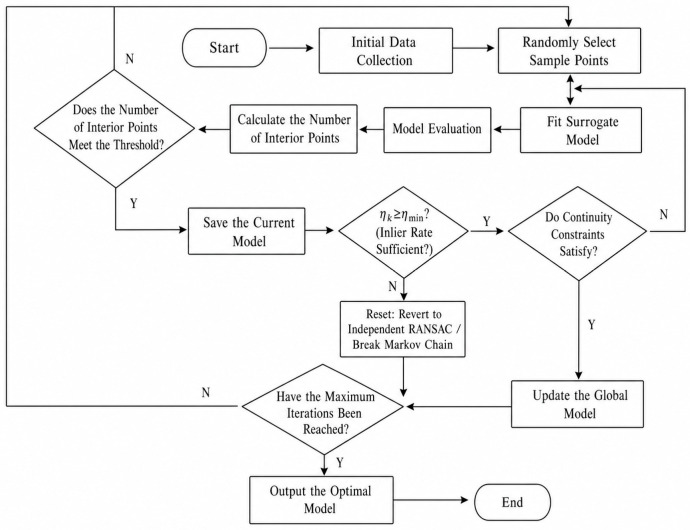
CC-RANSAC Algorithm Flowchart.

By maximizing this function, CC-RANSAC considers not only the consistency of the local point cloud but also the geometric continuity with the previous cross-section when fitting the current cross-section. This effectively suppresses the sudden parameter changes commonly observed in traditional independent cross-section fitting, thereby improving the stability of parameter recovery across continuous cross-sections while maintaining robustness. The overall solution workflow of CC-RANSAC is shown in Figure 9. Building upon RANSAC’s outlier-tolerance capabilities, this method introduces continuity constraints regarding the center position, major and minor axes, and rotation angle between adjacent cross-sections. It performs a joint scoring of candidate elliptical models, thereby enhancing the stability and engineering interpretability of continuous cross-section parameter extraction.

#### 2.4.4. Deformation Indicators

Based on the elliptical parameters of the continuous cross-section obtained through CC-RANSAC reconstruction, key indicators reflecting the lateral deformation state of the tunnel can be further calculated. This paper focuses on using the major axis, minor axis, and ovality as quantitative characterization parameters. Among these, ovality is defined as:

(11) $$c=\frac{a-b}{r}$$

where c denotes the ovality (ellipticity) of the cross-section, a and b represent the major and minor semi-axes of the measured ellipse, respectively, and r is the theoretical design radius of the shield tunnel.

Ellipticity characterizes the degree of deviation of the cross-section from an ideal circle and serves as a key indicator of asymmetric deformation and elliptical effects in shield-driven tunnels. For shield-driven tunnels in operation, an increase in the difference between the major and minor axes indicates significant convergence or expansion of the structure in a particular direction, suggesting that the structural condition may be approaching an abnormal state.

#### 2.4.5. Experimental Setup and Evaluation Metrics

To validate the effectiveness of the proposed method, this paper constructs a semantic segmentation dataset based on actual LiDAR point clouds collected from a shield tunnel of an operational subway line in Shanghai. The total number of points in the raw scan point cloud is approximately 40.44 million, covering straight sections, curved sections, and mixed conditions. Considering the large overall dataset size and the need for efficient training, this paper selected Section 70 as the core experimental sample for semantic segmentation, yielding a total of 303,819 points after preprocessing. Section 70 was chosen because it is a transitional segment that encompasses straight alignment, mild curvature, and a mixed structural zone within a continuous scan, and therefore contains the full range of point cloud characteristics—including density variation, occlusion patterns, and background category distribution—that are representative of the broader dataset. In addition, the six semantic categories present in Section 70 (power grid, wires, rails, pipes, bolt holes, and lining) reflect the complete auxiliary facility composition observed across the entire scanned tunnel. It should be further noted that the deformation extraction experiments in Section 3 are conducted on a separate, larger dataset spanning 80 ring segments (Groups 1–4), which explicitly covers straight sections, curved sections, and mixed conditions, thereby providing a broader validation basis beyond the single-section segmentation training sample. 

Based on the distribution of the tunnel’s primary structures and ancillary facilities, the point cloud was manually annotated in detail to establish six semantic labels: power grid, electrical wires, rails, pipelines, bolt holes, and lining. Among these, the lining comprises 228,311 points, accounting for 75.15%; the remaining categories collectively form the complex background point cloud. The number of samples and their proportions for each category in the dataset are shown in Table 2. To accommodate subsequent cross-section extraction tasks, the six-category results were further consolidated into two categories—“Lining” and “Background”—for engineering applications, as shown in Table 3.

The dataset is split into a training set and a validation set in an 8:2 ratio. The training set contains 243,055 points, while the validation set contains 60,764 points. The dataset covers typical tunnel point clouds under various mileage conditions and occlusion scenarios, enabling a thorough evaluation of the model’s fine-grained recognition capabilities and generalization performance in complex operating conditions.

The experimental setup described in this paper is shown in Table 4. Model training was performed in a Ubuntu environment, with the PWAT network implemented using Python and a deep learning framework, and accelerated on a GPU platform.

To comprehensively evaluate the performance of PWAT in point cloud semantic segmentation, this paper employs metrics such as Overall Accuracy (OA), Mean Intersection over Union (mIoU), Precision, Recall, and F1-score. Among these, OA reflects the model’s overall classification accuracy, while mIoU provides a more balanced assessment of segmentation performance across different classes—particularly for small-object categories—and is therefore more representative in this study. To evaluate the accuracy and stability of CC-RANSAC in cross-section geometric reconstruction, this paper employs metrics such as Mean Residual (MR), Standard Deviation (SD), Center Jump Rate (CJR), as well as Mean Absolute Error (MAE) and Root Mean Square Error (RMSE) obtained by comparing with total station measurements. Specifically, MR and SD are used to characterize the accuracy of the elliptical fit and the stability of the results; CJR is used to quantify whether non-physical geometric jumps exist between adjacent cross-sections; and MAE and RMSE are used to evaluate the consistency of the method’s fit with actual engineering monitoring values. Specifically, a Leica TS-series total station (with a nominal distance measurement accuracy of ±(1 mm + 1.5 ppm)) was employed to acquire reference measurements on 12 representative cross-sections within the study segment, with multiple characteristic points manually observed on the lining surface of each cross-section. The total station survey and the LiDAR scan were conducted within a short time interval to ensure that both datasets reflect the same structural state, thereby providing a reliable external benchmark for quantitatively evaluating the absolute accuracy of the proposed method.

## 3. Results

### 3.1. Semantic Segmentation Performance of PWAT

To validate the effectiveness of the PWAT network in the semantic segmentation of LiDAR point clouds in complex operational shield-driven tunnels, this paper conducts comparative experiments using a six-class point cloud dataset and selects PointNet and PointNet++ as baseline models. The evaluation metrics include overall accuracy (OA), mean intersection-over-union (mIoU), F1-score, and Kappa, as shown in Table 5.

The experimental results show that PWAT achieved the best results across all evaluation metrics, with an OA of 99.53%, an mIoU of 99.06%, an F1-score of 99.53%, and a Kappa coefficient of 0.98. Compared with PointNet++, PWAT achieved improvements of 0.62% and 2.81% in OA and mIoU, respectively, indicating that the proposed method outperforms the baseline model in overall segmentation performance.

Further results by category are shown in Table 6. PWAT demonstrates high recognition accuracy for all six target categories, with IoU values of 0.9874, 0.9917, 0.9939, 0.9852, 0.9895, and 0.9960 for power grids, wires, tracks, pipes, bolt holes, and lining, respectively. In particular, for small object categories such as power grids, pipelines, and bolt holes, the IoU values all exceed 98%, indicating that PWAT possesses strong fine-grained classification capabilities in complex tunnel scenarios.

To further validate the performance advantages of PWAT over newer point cloud segmentation models, and considering that subsequent lateral deformation extraction relies primarily on the accurate extraction of lining point clouds, this paper additionally presents a comparative analysis with DGCNN, RandLA-Net, and Point Transformer on the “lining–background” binary classification task. The results are shown in Table 7. It should be noted that the six-class classification results are primarily used to validate the model’s fine-grained semantic segmentation capabilities in complex tunnel scenarios, whereas the binary classification results more directly serve the engineering application requirements for subsequent cross-section extraction and deformation analysis.

The results show that PWAT continues to demonstrate superior performance in binary classification tasks, achieving an overall accuracy of 99.31%, an average intersection-over-union (IoU) of 98.64%, and IoUs of 99.47% and 97.81% for the lining and background classes, respectively. Compared to PointNet++, PWAT achieves improvements of 2.48% in Overall Accuracy (OA) and 4.47% in mean IoU (mIoU). Compared to DGCNN and RandLA-Net, PWAT also maintains a lead in overall accuracy and average IoU. Compared to Point Transformer, PWAT retains a certain advantage in accuracy while offering parameter sizes and inference times better suited for engineering applications. These results demonstrate that PWAT not only possesses strong small-object recognition capabilities in the six-class classification task but also exhibits excellent comprehensive performance in engineering tasks focused on lining extraction, providing higher-quality input data for subsequent continuous cross-section fitting. Since the extraction of lateral deformation is highly dependent on the integrity and cleanliness of the lining boundaries, these results also lay the foundation for the subsequent CC-RANSAC algorithm to obtain stable cross-section parameters.

### 3.2. Further Analysis of PWAT

#### 3.2.1. Ablation Study

To justify the architectural complexity of PWAT, a progressive ablation study was conducted by incrementally adding three modules to the PointNet++ baseline: the k-NN-based Local Feature Extraction module (LFE), the multi-head self-attention with geometric position encoding (ATT), and the Weight-Adaptive Module (WAM), which together constitute the complete PWAT network. All configurations were trained five times under identical settings (learning rate 0.001, batch size 4, 200 epochs, Adam optimizer) and the mean result was reported. Overall performance results are shown in Table 8, and per-category IoU contributions are shown in Table 9.

As shown in Table 8, each module addition yields a consistent and progressive improvement across all metrics, with mIoU increasing from 96.25% (Baseline) to 99.06% (full PWAT). The incremental gains follow a decreasing trend (+0.87%, +0.77%, +0.81%), consistent with the progressive refinement of the model as each module addresses a specific limitation of the previous configuration.

Table 9 further reveals that improvements are most pronounced for small and structurally complex categories such as power grids, pipes, and bolt holes, confirming that LFE primarily strengthens small-object detection through density-adaptive neighborhood construction, ATT enhances inter-class feature discriminability at complex and blurred boundaries, and WAM refines boundary accuracy and overall model robustness. The results collectively demonstrate that each module contributes distinctly and that their combination produces a clear synergistic benefit, fully justifying the design of the PWAT architecture.

#### 3.2.2. Generalization Analysis Across Tunnel Conditions

To evaluate the generalization capability of PWAT under different operational conditions, cross-condition experiments were conducted using two 150 m segments of the same Shanghai metro tunnel: a straight section (K3 + 150 − K3 + 300) and a curved section (K5 + 420 − K5 + 570, curvature radius R = 350 m). After preprocessing, the straight and curved datasets contain 312,456 and 298,734 points, respectively. Compared to the straight section, the curved section exhibits approximately 15% greater density non-uniformity and 23% more locally occluded regions due to segment misalignment and joint opening effects under curvature. Three experimental groups were defined: Exp-1 (Straight→Curved), trained on the straight section and tested on the curved section; Exp-2 (Curved→Straight), trained on the curved section and tested on the straight section; and Exp-3 (Mixed), trained and tested on a combined dataset serving as a performance upper bound.

Overall performance results are shown in Table 10. PWAT consistently outperforms PointNet++ across all three conditions, achieving mIoU values of 73.6%, 75.1%, and 82.3% in Exp-1, Exp-2, and Exp-3, respectively, with OA values of 92.4%, 93.0%, and 94.8%. The mIoU degradation from mixed training to cross-condition testing is 8.7% for PWAT and 8.9% for PointNet++, indicating that PWAT’s greater architectural complexity does not introduce disproportionate overfitting to any single operational condition.

Per-category IoU results are shown in Table 11. For the lining class, PWAT maintains IoU values above 84% in all cross-condition settings, reflecting the robustness of geometric position encoding for large-scale cylindrical structures. For small and geometrically sensitive categories, the improvements are more pronounced: PWAT outperforms PointNet++ by 27.5% on bolt holes and 13.7% on wires in Exp-1, demonstrating that k-NN adaptive sampling and geometry-constrained attention effectively preserve fine-grained features under density-varying curved tunnel conditions. Mixed training (Exp-3) further improves performance across all categories by 8–12%, suggesting that incorporating diverse operational conditions into the training set is beneficial when the target deployment environment is known in advance.

#### 3.2.3. Analysis of the Impact of Key PWAT Parameters

To validate the rationality of PWAT’s key architectural design and determine the optimal parameter configuration for the model, this paper conducts a sensitivity analysis on the number of k-nearest neighbors, the number of attention heads, and the voxel downsampling resolution, drawing on the parameter analysis experiments in the original paper.

First, the number of k-nearest neighbors directly affects the local neighborhood range and feature extraction performance. As shown in Table 12, as the number of k-nearest neighbors increases, the model’s performance generally follows an initial upward trend followed by a decline. When k = 32, PWAT achieved the best results, with mIoU, OA, and F1-score reaching 99.06%, 99.53%, and 99.53%, respectively. As k continued to increase, model performance declined slightly, while training time and GPU memory usage increased significantly.

Second, the number of attention heads affects the modeling capability of the self-attention module for different feature subspaces. As shown in Table 13, model performance improves continuously as the number of heads increases from 1 to 8; however, when the number of heads is further increased to 16, performance declines slightly while computational cost rises significantly. Balancing accuracy and resource consumption, we selected 8 attention heads as the final configuration in this paper.

As shown in Table 14, voxel resolution affects both segmentation accuracy and processing efficiency. The model achieves optimal performance at a resolution of 0.03 m; as the resolution continues to increase, both mIoU and OA decline. Under the configuration of k = 32, 8 attention heads, and a voxel resolution of 0.03 m, PWAT demonstrates the most balanced IoU performance across all categories.

Based on the above results, this paper ultimately determines the optimal parameter configuration for PWAT as follows: k = 32, 8 attention heads, and a voxel downsampling resolution of 0.03 m. This configuration achieves a good balance between segmentation accuracy and computational efficiency. The sensitivity results carry clear engineering interpretations. For the k-NN neighborhood size, performance peaks at k = 32 and degrades at larger values (k = 64, 128) because an overly large neighborhood incorporates an increasing proportion of geometrically dissimilar points from adjacent structural categories—such as bolt hole edges being contaminated by lining surface points—which dilutes the local feature representation. At smaller values (k = 8, 16), the neighborhood is insufficient to capture the complete local geometry of thin linear structures such as wires and power grids, leading to feature under-sampling. For the number of attention heads, fewer heads (1–2) lack the capacity to model multiple geometric relationships simultaneously, while 16 heads introduce parameter redundancy that exceeds what the limited training data can support, resulting in slight overfitting. Eight heads provide the best balance, allowing different heads to specialize in distinct geometric aspects such as boundary curvature, spatial topology, and inter-category contrast.

### 3.3. Visual Comparison of Segmentation Results

**Figure 10:**
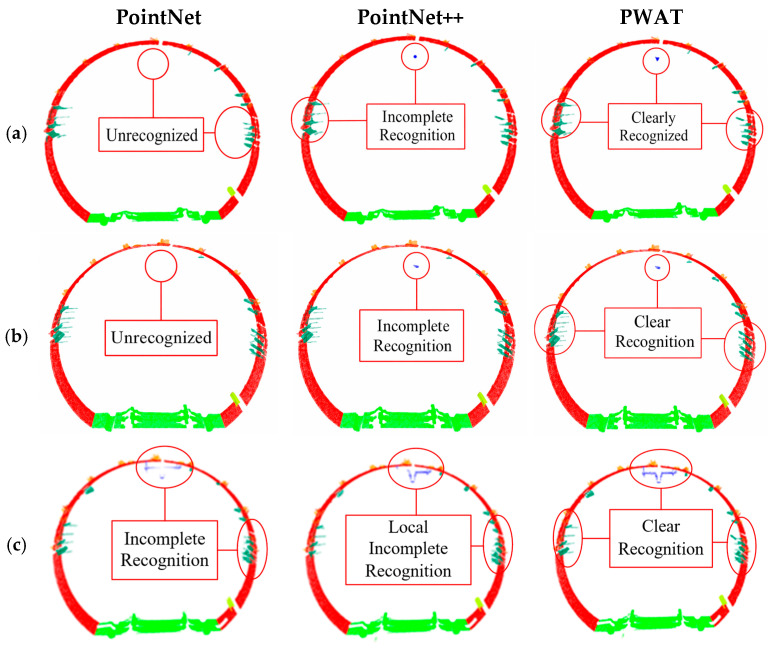

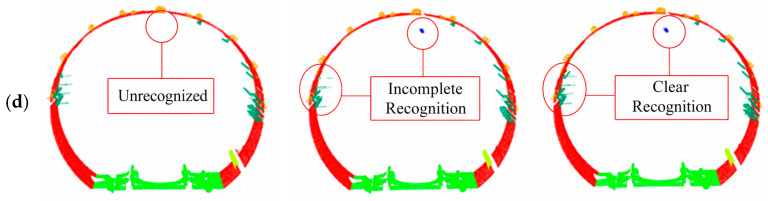
Analysis of Visualization Results from the PWAT Model for Different Point Cloud Datasets. (**a**–**d**) show tunnel segments from different sections. Red indicates the tunnel lining, orange indicates bolt holes, dark green indicates electrical wiring, light green indicates rails, blue indicates the power supply network, and yellow indicates pipes.

To provide a visual comparison of the segmentation results from different methods, this paper selected four typical pipe segment regions and visualized the semantic segmentation results of PointNet, PointNet++, and PWAT, as shown in Figure 10.

As shown in Figure 10, PWAT produces more complete lining contours and clearer class boundaries across different tunnel sections. Compared to PointNet and PointNet++, PWAT exhibits fewer misclassifications and omissions near joints, in the vicinity of small components, and in locally occluded areas, and the boundaries between ancillary facilities and the lining are more continuous. Even in areas with significant variations in point density, PWAT maintains relatively stable segmentation results.

Overall, the point cloud boundaries of the lining generated by PWAT are more complete and contain fewer background artifacts, providing higher-quality input data for subsequent continuous cross-section fitting.

### 3.4. Performance of CC-RANSAC in Continuous Ellipse Fitting

**Figure 11:**
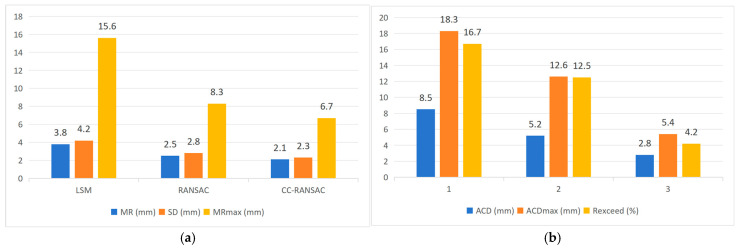
Comparison of Performance Among Different Fitting Algorithms: (**a**) Comparison of Fitting Accuracy Metrics; (**b**) Comparison of Continuity Metrics.

Based on the PWAT-segmented lining point cloud, this paper employs CC-RANSAC for continuous cross-sectional elliptical fitting and compares it with the Least Squares (LS) method and traditional RANSAC. The results are shown in Figure 11. Compared with the two alternative methods, CC-RANSAC performs better in terms of mean residual, standard deviation of residuals, and continuity metrics.

**Figure 12:**
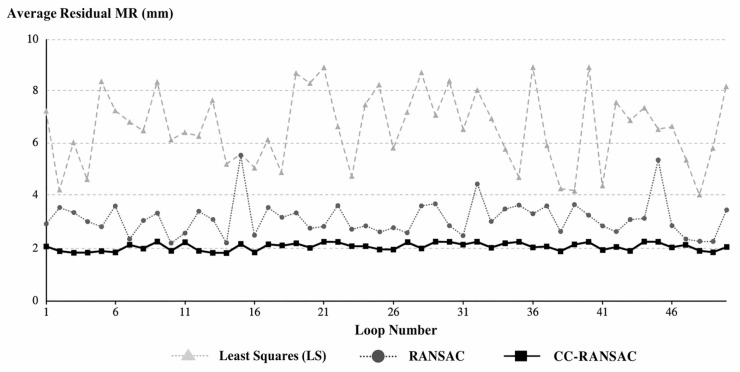
Comparison of Average Residuals Across 50 Consecutive Rings Under Different Algorithms.

Furthermore, Figure 12 shows the trend in the average residuals over 50 consecutive cycles. The least-squares method exhibits a relatively large overall error with significant fluctuations, while traditional RANSAC reduces the residuals but still shows noticeable fluctuations in local sections. In contrast, the residual curve for CC-RANSAC is smoother overall and remains at a lower level.

The results above indicate that CC-RANSAC achieves more stable parameter recovery in the fitting of consecutive cross-sections.

### 3.5. Field Validation of CC-RANSAC

To verify the applicability of CC-RANSAC to point cloud data from actual operational shield-driven tunnels, a two-level validation strategy is adopted: first, the three fitting algorithms (LS, traditional RANSAC, and CC-RANSAC) are compared on the LiDAR point cloud itself in terms of internal fitting quality and inter-section continuity; then, total station measurements are introduced as an independent external benchmark to further evaluate the absolute accuracy of each algorithm against the true structural state. The internal performance comparison of the different methods is shown in Table 15.

**Figure 13:**
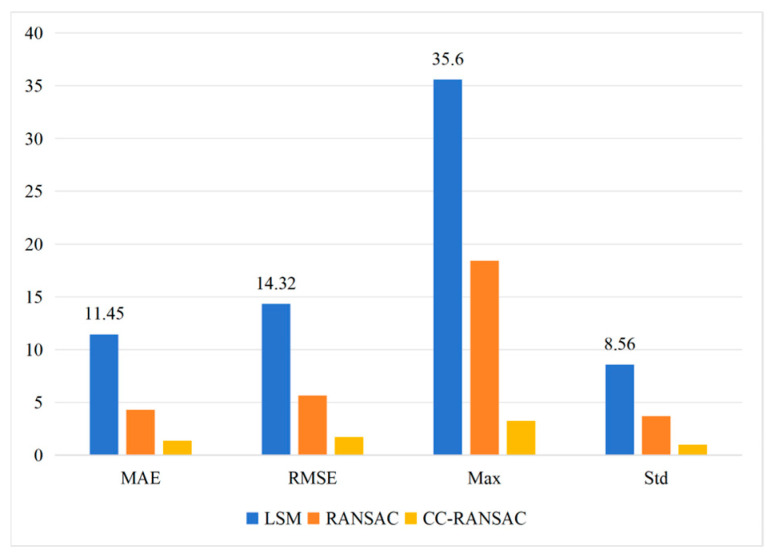
Comparison of Differences Between Various Algorithms and Actual Measured Values.

As shown in Table 11, CC-RANSAC outperforms the two comparison methods in terms of metrics such as mean residual, standard deviation, maximum residual, and center variation rate. Specifically, the mean residual is 2.0 mm, the standard deviation is 3.0 mm, the maximum residual is 10.5 mm, and the center variation rate is 4.2%. Compared with traditional RANSAC, CC-RANSAC shows significant improvements in both continuity and stability metrics, although the processing time per cross-section increases from 2.31 s to 2.84 s. To further verify the absolute accuracy and engineering reliability of the proposed method, the extraction results were compared against total station measurements, which are adopted here as the ground-truth reference owing to their high single-point accuracy and widespread acceptance in tunnel monitoring practice. A total of 12 cross-sections with retained total station observation points from the operational tunnel were selected for this comparative analysis, and for each cross-section the fitted ellipse contour was evaluated against the corresponding total station points to compute MAE and RMSE. The error statistics of the different fitting algorithms relative to the total station measurements are summarized in Table 16 and visualized in Figure 13.

As shown in Table 14, the Least Squares method suffered severely from the interference of ancillary facilities on the tunnel wall, resulting in the largest overall error with a Mean Absolute Error (MAE) of 11.45 mm and a maximum deviation of up to 35.60 mm. Although the traditional RANSAC algorithm effectively eliminated some noise and reduced the MAE to 4.28 mm, it still easily fell into local optima when facing severe occlusions or missing local point clouds, leaving a maximum deviation of 18.42 mm and a standard deviation of 3.68 mm. Its robustness remained insufficient for high-precision requirements.

In stark contrast, the proposed CC-RANSAC algorithm outperformed the other methods across all evaluation metrics. By introducing continuity constraints, the MAE was reduced to only 1.35 mm, and the Root Mean Square Error (RMSE) was as low as 1.68 mm. Furthermore, the maximum deviation was strictly controlled within 3.24 mm, with a standard deviation of just 0.98 mm. This comprehensive comparison with the widely accepted total station method strongly demonstrates that the proposed CC-RANSAC algorithm can effectively overcome the interference of complex operational conditions, acquiring geometric parameters that are highly consistent with the true physical state of the tunnel.

**Figure 14:**
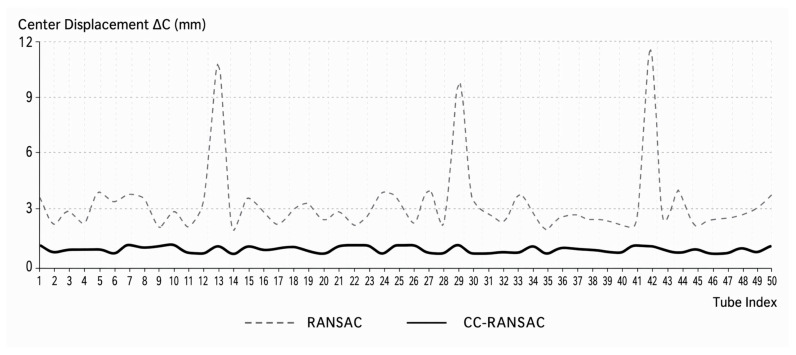
Distribution of center-of-gravity offsets for 50 consecutive cross-sections.

Figure 14 further shows that traditional RANSAC exhibits relatively pronounced peaks of center shift in some sections, whereas CC-RANSAC exhibits smoother overall variation.

Overall, CC-RANSAC is capable of achieving high-precision, stable geometric reconstruction of continuous cross-sections in point cloud data from real-world operational tunnels.

### 3.6. Deformation Characterization Results

**Figure 15:**
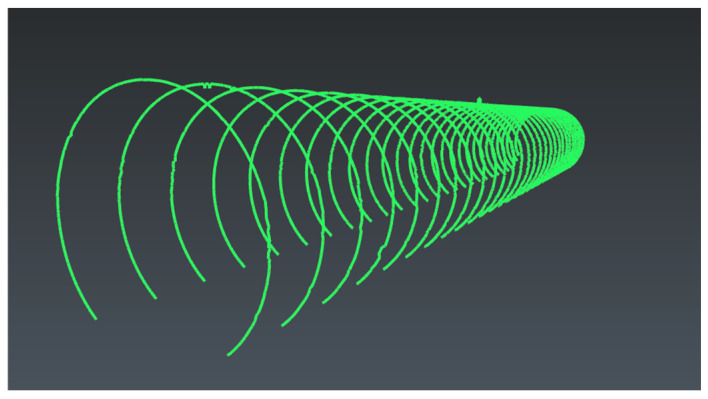
Visualization of Elliptical Fitting for Continuous Cross-Sections.

Based on the results of continuous elliptical fitting, this paper further extracts parameters such as the center of the ellipse, the major and minor axes, and the ellipticity to characterize the geometric state of the shield tunnel cross-section. The results of elliptical fitting for a typical continuous cross-section are shown in Figure 15. It can be seen that the fitted ellipse varies continuously along the tunnel axis, with no obvious local drift or abrupt changes observed overall.

**Figure 16:**
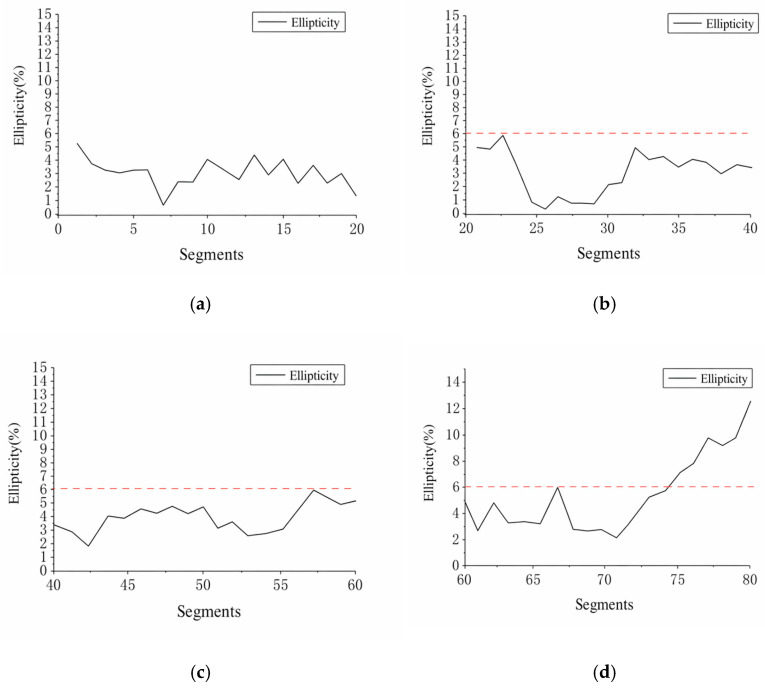
Comparative Analysis Results of Four Sets of Validation Data.: (**a**) Segments numbered 0–20; (**b**) Segments numbered 20–40; (**c**) Segments numbered 40–60; (**d**) Segments numbered 60–80. The red dashed line represents the 6‰ standard line for ellipticity.

To analyze the lateral deformation characteristics of different sections, this study conducted a statistical summary of four representative test sections. The results are shown in Table 17. The results indicate that Section 1 was the most stable overall, with the ellipticity of all ring sections below 6‰; Groups 2 and 3 each had one ring segment exceeding the warning threshold, indicating localized anomalies; Group 4 had the highest number of segments exceeding the threshold, with a total of 7 (35.0%), and the maximum ellipticity reached 12.3‰, demonstrating a more pronounced tendency toward elliptical deformation. An overall comparison is shown in Figure 16.

Overall, the average ellipticity of the four sections was 2.71‰, 3.27‰, 4.00‰, and 5.53‰, respectively, showing a gradual increase. These results indicate that the method described in this paper can extract deformation parameters from continuous cross-sections and effectively identify deformation differences between different sections as well as the locations of local anomalies.

## 4. Discussion

The proposed method provides an integrated LiDAR-based framework for lateral deformation extraction in operational shield tunnels. It focuses on real working conditions with complex facilities, occlusions, noise, and uneven point density. Compared with traditional discrete measurement methods, this framework can process raw point clouds automatically and output continuous deformation indicators for structural health assessment.

In terms of semantic segmentation, the PWAT network is designed to fit tunnel point cloud features. It uses k-NN adaptive sampling, geometric position encoding, and geometrically constrained self-attention. These improvements help the network distinguish linings from small facilities such as wires, pipes, and bolt holes, even in areas with uneven density or weak boundaries. High segmentation accuracy ensures that the lining point cloud is clean and complete, which is essential for stable ellipse fitting and deformation calculation.

For continuous section reconstruction, the key improvement of CC-RANSAC is introducing continuity constraints between adjacent sections. Traditional independent fitting easily produces unreasonable parameter jumps caused by local noise or missing data. CC-RANSAC limits sudden changes in ellipse center, axes, and rotation angle. The recovered parameters follow the natural gradual change in the tunnel structure. For engineering monitoring, this stability is more meaningful than single-section accuracy, because operation and maintenance usually focus on continuous deformation trends and abnormal regions.

From the deformation results, the proposed method can effectively reflect the structural state of different tunnel sections. Among the four tested groups, sections 1–3 show relatively stable deformation, while Section 4 has higher average ovality and more segments exceeding the threshold. This shows that the method can distinguish section-level deformation differences and support abnormal location detection and risk assessment.

Nevertheless, this study has certain limitations. First, the method is verified on one metro tunnel line. Further testing on more tunnel types, scanning devices, and severe occlusion scenarios is needed to improve generalization. For cross-sections with severe occlusion and an insufficient number of valid lining points, quality marking or elimination should be performed in practical engineering applications. In the future, we will further improve data integrity by combining multi-station scanning, multi-temporal point clouds, and more robust occlusion compensation strategies. Second, the current framework uses an elliptical model, which can represent global deformation but may not describe local distortions such as joint dislocation and local convergence in detail. Third, the current method relies on single-epoch point clouds and compares them with theoretical design values. It is acknowledged that initial construction deviations (as-built vs. design geometry) exist. In future practical applications, acquiring the initial post-construction point cloud as a baseline (zero-epoch) for multi-temporal comparison will be necessary to completely separate operational deformation from construction errors. Furthermore, with continued advances in scanning hardware and edge computing, there exists potential for extending the proposed pipeline toward real-time or near-real-time structural health monitoring, which would further enhance its practical value for operational tunnel management.

Overall, the proposed PWAT + CC-RANSAC framework achieves accurate, stable, and automatic lateral deformation extraction from LiDAR point clouds. It supports intelligent health monitoring for operational shield tunnels and can be integrated into digital twin and automatic inspection systems.

## 5. Conclusions

This paper proposes a high-precision lateral deformation extraction method using LiDAR point clouds for operational shield tunnels. A PWAT segmentation network and a CC-RANSAC continuous fitting algorithm are combined to build an integrated workflow: point cloud preprocessing, lining extraction, continuous geometric recovery, and deformation evaluation. The method is verified using measured data from a Shanghai metro tunnel. The main conclusions are as follows:

1. The proposed PWAT network achieves high-precision semantic segmentation of point clouds from complex operational tunnels. By incorporating k-nearest-neighbor adaptive sampling, geometric position encoding, and a multi-head self-attention mechanism with geometric constraints, the model enhances its ability to distinguish between the lining and ancillary facilities. It achieves high overall accuracy and mean intersection-over-union (mIoU) in a six-class classification task, demonstrating particularly good recognition performance for small-target categories such as power grids, pipelines, and bolt holes.
2. The proposed CC-RANSAC method enables stable recovery of elliptical parameters across consecutive cross-sections. Compared to traditional least-squares methods and RANSAC, this approach retains the ability to resist outliers while significantly reducing parameter jumps by introducing continuity constraints between adjacent cross-sections, thereby improving the stability and physical consistency of geometric recovery across consecutive cross-sections.
3. The lateral deformation parameters extracted using the proposed method demonstrate good engineering applicability. Comparison with total station measurement results indicates that the method can achieve millimeter-level accuracy in cross-section parameter recovery within complex operational tunnel environments; section-by-section statistical analysis further demonstrates that the method can effectively identify deformation differences across different sections and locate local anomalies.

Overall, the method proposed in this paper achieves automated conversion from complex LiDAR point clouds to tunnel lateral deformation metrics, providing technical support for structural health monitoring and intelligent operation and maintenance of operational shield-driven tunnels. Future research could focus on multi-temporal point cloud analysis, cross-scenario generalization, and methods for characterizing more complex cross-sectional geometries.

## Figures and Tables

**Table 1:** Summary and Comparison of Downsampling Methods.

Resolution	Number of Point Clouds After Compression (Units)	Time (Seconds)	Compression Ratio (%)
Initial point cloud	40,365,512	-	-
0.01	908,300	82	97.75
0.02	388,487	74	99.04
0.03	115,704	52	99.71
0.05	14,841	36	99.96
0.08	5475	18	99.99
0.10	1371	11	99.99

**Table 2:** Number of Classification Points for Tunnel Auxiliary Facilities.

Category	1-Power Grid	2-Wires	3-Rails	4-Pipes	5-Bolt Holes	6-Lining
Units/points	3518	15,078	47,692	3106	6114	228,311
percentage	1.16%	4.96%	15.70%	1.02%	2.01%	75.15%

**Table 3:** Number of classification points in Tunnel 2.

Category	Number of Points	Percentage	Training Set	Validation Set
lining	228,311	75.2%	182,649	45,662
Background	75,508	24.8%	60,406	15,102
Total	303,819	100%	243,055	60,764

**Table 4:** Training Environment.

	Configuration	Model
Hardware platform	CPU	Intel Core i7-11700, 2.50 GHz
	Graphics card	NVIDIA GeForce RTX 3060
	Memory	16.0 GB
Software platform	operating system	Ubuntu 18.04
	Programming languages	Python 3.9
	Deep learning frameworks	TensorFlow 1.13.1
	Third-party libraries	Open3D 0.13.0

**Table 5:** Comparative Analysis of Model Accuracy.

Model	OA (%)	mIoU (%)	F1-Score	Kappa
PointNet	98.35	87.36	93.08	0.95
PointNet++	98.91	96.25	98.08	0.97
PWAT	99.53	99.06	99.53	0.98

**Table 6:** IoU scores across six categories.

Model	1-Power Grid	2-Wires	3-Rails	4-Pipes	5-Bolt Holes	6-Lining
PointNet	0.8060	0.8960	0.9410	0.7611	0.8513	0.9862
PointNet++	0.9512	0.9667	0.9736	0.9427	0.9598	0.9813
PWAT	0.9874	0.9917	0.9939	0.9852	0.9895	0.9960

**Table 7:** Comparison of the PWAT Method with Mainstream Models in Lining-Background Binary Classification Accuracy.

Model	OA (%)	mIoU (%)	Lining IoU (%)	Background IoU (%)	Number of Participants (M)	Training Time (s)
PointNet	94.52	91.38	96.73	86.02	3.5	78
PointNet++	96.83	94.17	97.95	90.38	1.5	125
DGCNN	97.41	95.24	98.32	92.15	1.8	142
RandLA-Net	97.89	95.76	98.64	92.87	1.2	95
PointTransformer	98.15	96.42	98.89	93.94	7.8	256
PWAT	99.31	98.64	99.47	97.81	2.3	108

**Table 8:** Overall performance comparison across ablation configurations.

Configuration	mIoU (%)	OA (%)	F1-Score (%)	Kappa
Baseline (PointNet++)	96.25	98.91	98.08	0.971
Exp-1 (+LFE)	97.12	99.08	98.61	0.976
Exp-2 (+ATT)	97.89	99.24	98.97	0.981
PWAT (+WAM, full)	99.06	99.53	99.53	0.987

**Table 9:** Per-category IoU (%) across ablation configurations.

Configuration	1-Power Grid	2-Wires	3-Rails	4-Pipes	5-Bolt Holes	6-Lining
Baseline	95.12	96.67	97.36	94.27	95.98	98.13
Exp-1	96.31	97.42	97.89	95.84	96.72	98.54
Exp-2	97.15	98.03	98.46	96.93	97.58	98.87
PWAT	98.74	99.17	99.39	98.52	98.95	99.60

**Table 10:** Overall performance across working conditions.

Model	Exp-1: Straight→Curved		Exp-2: Curved→Straight		Exp-3: Mixed Training	
	mIoU (%)	OA (%)	mIoU (%)	OA (%)	mIoU (%)	OA (%)
PointNet++	58.4	88.6	61.2	89.4	67.3	91.5
PWAT	73.6	92.4	75.1	93.0	82.3	94.8

Note: Exp-1: trained on straight section, tested on curved section. Exp-2: trained on curved section, tested on straight section. Exp-3: mixed training as performance upper bound.

**Table 11:** Per-category IoU (%) across working conditions.

Category	PointNet++ S→C (%)	PWAT S→C (%)	PointNet++ C→S (%)	PWAT C→S (%)	PointNet++ Mixed (%)	PWAT Mixed (%)
1-Power grid	72.4	84.7	75.1	86.3	81.2	91.5
2-Wires	68.3	79.5	70.6	81.2	76.8	86.3
3-Rails	61.5	73.2	63.8	75.6	69.4	82.1
4-Pipes	54.2	68.9	56.7	70.4	62.5	77.8
5-Bolt holes	48.6	62.3	51.3	64.8	57.1	71.4
6-Lining	45.3	72.8	49.8	74.5	56.3	84.7

Note: S→C: Straight→Curved; C→S: Curved→Straight.

**Table 12:** Analysis of the Impact of the Number of Nearest Neighbors on the Performance of Each Model Module.

Number of Nearest Neighbors	mIoU (%)	OA (%)	F1-Score (%)	Training Time (s)	GPU Memory (GB)	Performance Tier
8	95.42	98.85	96.12	42	4.2	Average
16	97.28	99.15	97.45	52	4.8	Good
32	99.06	99.53	99.53	68	5.8	Excellent
64	98.73	99.41	98.95	98	7.2	Good
128	97.85	99.22	98.01	156	10.1	Average

**Table 13:** Analysis of the Impact of the Number of Attention Heads on the Performance of Each Model Module.

Number of Attention Heads	mIoU (%)	OA (%)	F1-Score (%)	Number of Participants (M)	GPU Memory (GB)	Feature Representation Capability
1	96.14	98.92	96.45	2.1	3.2	Weak
2	97.56	99.18	97.78	2.8	4.1	Average
4	98.34	99.35	98.56	3.2	4.8	Good
8	99.06	99.53	99.53	4.1	5.8	Excellent
16	98.78	99.44	98.89	6.8	9.6	Excessive

**Table 14:** Analysis of the Impact of Voxel Resolution on the Performance of Various Model Modules.

Voxel Resolution (m)	Number of Points	Compression Ratio (%)	mIoU (%)	OA (%)	Training Time (s)
0.01	908,300	97.75	98.42	99.38	180
0.02	388,487	99.04	98.89	99.48	120
0.03	115,704	99.71	99.06	99.53	68
0.05	14,841	99.96	97.73	99.21	36
0.08	5475	99.99	95.68	98.85	18

**Table 15:** Performance Comparison of Different Elliptical Fitting Algorithms.

Evaluation Criteria	LS	RANSAC	CC-RANSAC	Magnitude of Improvement
Accuracy metrics				
MR (mm)	5.2	2.3	2.0	↑ 13.0%
SD (mm)	9.1	3.8	3.0	↑ 21.1%
MRmax (mm)	42.3	13.7	10.5	↑ 23.4%
Continuity indicators				
CJR (%)	37.5	29.2	4.2	↑ 85.6%
Efficiency metrics				
Processing time per cross-section (s)	0.08	2.31	2.84	↓ 22.9%
Average number of iterations	1	158	187	↓ 18.4%

Note: ↑ indicates an improvement in performance; ↓ indicates a decrease in performance (processing time); Improvement rate = (Method described in this paper—Comparison method)/Comparison method × 100%.

**Table 16:** Error Statistics of Different Algorithms Relative to Total Station Measurements.

Algorithm	MAE (mm)	RMSE (mm)	MDmax (mm)	SD (mm)
LS	11.45	-	35.60	-
RANSAC	4.28	-	18.42	3.68
CC-RANSAC	1.35	1.68	3.24	0.98

**Table 17:** Summary of Statistical Results for Lateral deformation in Four Validation Sections.

Group	Group 1	Group 2	Group 3	Group 4	Total
Ring	1–20	21–40	41–60	61–80	1–80
Ring Segments	20	20	20	20	80
Major Axis Range (m)	5.4731–5.5154	5.4990–5.5164	5.5038–5.5208	5.5080–5.5355	5.4731–5.5355
Minor Axis Range (m)	5.4460–5.4977	5.4789–5.5013	5.4851–5.4968	5.4678–5.4980	5.4460–5.5013
Ellipticity Range (‰)	0.3–4.9	0.2–6.5	1.7–6.1	2.2–12.3	0.2–12.3
Average Ellipticity (‰)	2.71	3.27	4.00	5.53	3.88
Percentage Above Threshold (%)	0	1	1	7	9
Maximum Deviation (mm)	26.9	16.4	20.8	35.5	35.5

## Data Availability

The data presented in this study are available on request from the corresponding author.
